# Supplementary material for: The Virus-Induced Transcription Factor SHE1 Interacts with and Regulates Expression of the Inhibitor of Virus Replication (IVR) in N Gene Tobacco
Source: Viruses. 2022 Dec 24;15(1):59. doi: 10.3390/v15010059 (PMC9864551; doi:10.3390/v15010059)
Supplement: Supplementary file 1 [file viruses-15-00059-s001.zip › viruses-2088419-supplementary.pdf]

Supplementary Tables and Figures

The virus–induced transcription factor SHE1 interacts with and regulates the expression of the inhibitor of virus replication (IVR) in *N* gene tobacco

Ju-Yeon Yoon [1,2*], Eseul Baek [1], Mira Kim [1] and Peter Palukaitis [1]

[1] Department of Horticulture Sciences, Seoul Women's University, Seoul 01797, Republic of Korea

[2] Department of Plant Protection and Quarantine, College of Agriculture and Life Sciences, Jeonbuk National University, Jeonju-si 54896, Republic of Korea

* Correspondence: juyeon74@gmail.com

Table S1. Oligonucleotide primers used in reverse-transcription-polymerase chain reaction amplification for the yeast two-hybrid assay

Primers[a]	Orientation	Sequences[b]	Target size (bp)
SHE1-For	sense	5'-GGGG*ACAAGTTTGTACAAAAAAGCAGGCT*TC ATGTCAAGTAACTCAAGC-3'	705
SHE1-Rev	antisense	5'-GGGG*ACCACTTTGTACAAGAAAGCTGGGTC* GTCCCTTCGACACGAATGTTGT-3'	
SHE1-555-Rev	antisense	5'-GGGG*ACCACTTTGTACAAGAAAGCTGGGTC* AGTAGCTTTTAGAACAGC-3'	555
SHE1-405-Rev	antisense	5'-GGGG*ACCACTTTGTACAAGAAAGCTGGGTC* TTCTTCAGCTGTATCAAA-3'	405
SHE1-255-Rev	antisense	5'-GGGG*ACCACTTTGTACAAGAAAGCTGGGTC* TACCTCTTCTAATTCTTG-3'	255
IVR-For	sense	5'-GGGG*ACAAGTTTGTACAAAAAAGCAGGCT*TC ATGAATCGACCTGAAGCTG-3'	600
IVR-Rev	antisense	5'-GGGG*ACCACTTTGTACAAGAAAGCTGGGTC* TAAAAGCTCAGCCTCTT-3'	
IVR-151-For	sense	5'-GGGG*ACAAGTTTGTACAAAAAAGCAGGCT*TC **ATG**CCACAGTCAGCAAAA-3'	450
IVR-301-For	sense	5'-GGGG*ACAAGTTTGTACAAAAAAGCAGGCT*TC **ATG**GTCATTGAAGGTCGAACT-3'	300

[a] Primers for SHE1 and IVR were designed based on the nucleotide sequences of SHE1 (AY655738, NCBI) and IVR (AJ009684).

[b] Gateway introduction sequences are shown in italics and underlined, with the ATG translation initiation codon (in bold) added to assure protein expression from IVR-151-For and IVR-301-For.

Table S2. Primers used for detection of viruses and plant mRNAs SHE1, IVR and EF1α

RNAs	Primers	Sequences	Product size (bp)
TMV-U1	TMV-For	5’-ATGTCTTACAGTATCACTAC-3’	523
	TMV-Rev	5’-TCAAGTTGCAGACCAGAGG-3’	
PVX-Kr	PVX-For	5’-ATGTCAGCACCAGCTAGCACA-3’	711
	PVX-Rev	5’-TTATGGCGGGAGAGTGAC-3’	
Fny-CMV	CMV-For	5’-GAGAGGATCCATGGACAAATCTGAATCAA-3’	657
	CMV-Rev	5’-GAGAGAGCTCGACTGGGAGCACTCCAGAT-3’	
PVY-O	PVY-For	5’-TTGACTCTTATGAAGTACACCATCA-3’	807
	PVY-Rev	5’-GTAAATACTTATATATCGTCCGGAG-3’	
SHE1	SHE1-For	5’-ATGTCAAGTAACTCAAGCCCAC-3’	705
	SHE1-Rev	5’-TCAGTCCCTTCGACACGAATGT-3’	
IVR	IVR-For	5’-ATGAATCGACCTGAAGCTGC-3’	600
	IVR-Rev	5’-TCATAAAAGCTCAGCCTC-3’	
EF1α	EF1α-For	5’-CTGACTGTGCTGTCCTGATTAT-3’	676
	EF1α-Rev	5’-TGATGACTTGGGAGGTAAAGCT-3’	

Table S3. Primers and vector information used for Bimolecular Fluorescence Complementation and Duolink *in situ* Proximity Ligation Assay

Targets	Primers[a,b]	Size (bp)	Destination vectors	
			BiFC	Duolink *in situ* PLA
SHE1	For: 5’-GGGG*ACAAGTT-TGTACAAAAAAGCAGGCT*<br>CCATGTCAAGTAACTCAAGCCCA-3’<br>Rev: 5’-GGGG*ACCACTTTGTACAAGAAAGCTGGGTC*<br>CAATGAAGTCATTAAAAG-3’	705	pDEST-^{GW}VYNE	pGWB412
IVR	For: 5’-GGGG*ACAAGTTTGTACAAAAAAGCAGGCT*<br>TCATGAATCGACCTGAAGCTG-3’<br>Rev: 5’-GGGG*ACCACTTTGTACAAGAAAGCTGGGTC*<br>TAAAAGCTCAGCCTCTT-3’	600	p(MAS)DEST-^{GW}SCYCE(R)	pGWB415

[a] Primers for SHE1 and IVR were designed based on the nucleotide sequences of SHE1 (AY655738) and IVR (AJ009684).

[b] Gateway sequences are in italics and underlined.

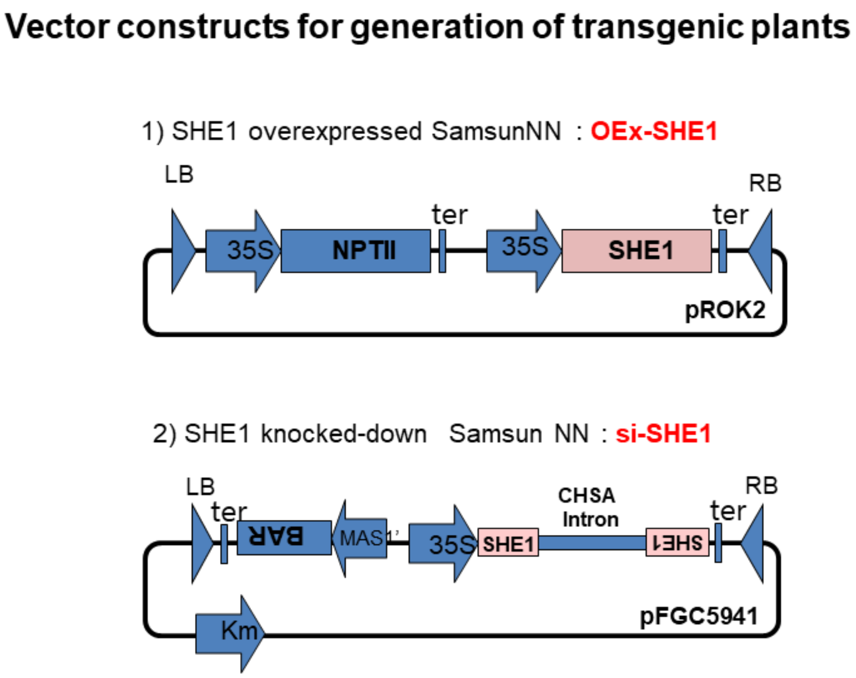

Figure S1. Plant transformation vectors constructed for overexpression of SHE1 (1) or silencing of expression of SHE1 (2).

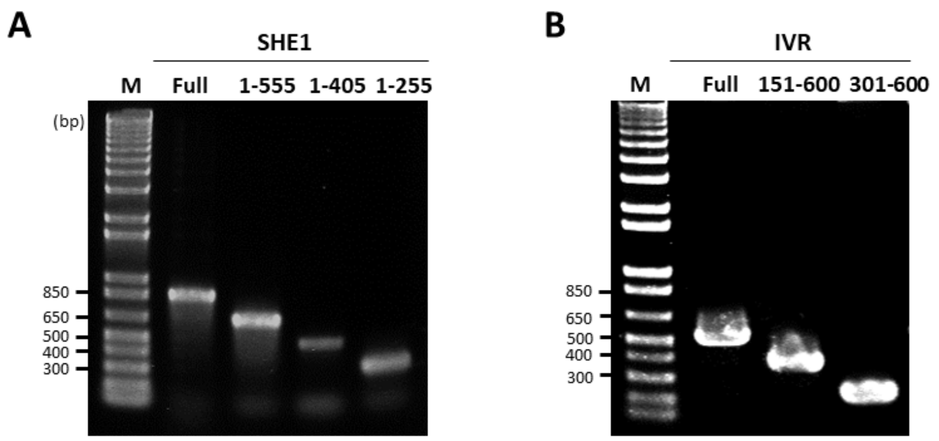

Figure S2. Amplification of the full-length and partial genes of (A) SHE1 and (B) IVR by RT-PCR for use in the yeast two-hybrid assay, and analysis of the RT-PCR products by agarose gel electrophoresis. M = 1 kb plus DNA Ladder (Invitrogen, Carlsbad, CA, USA).

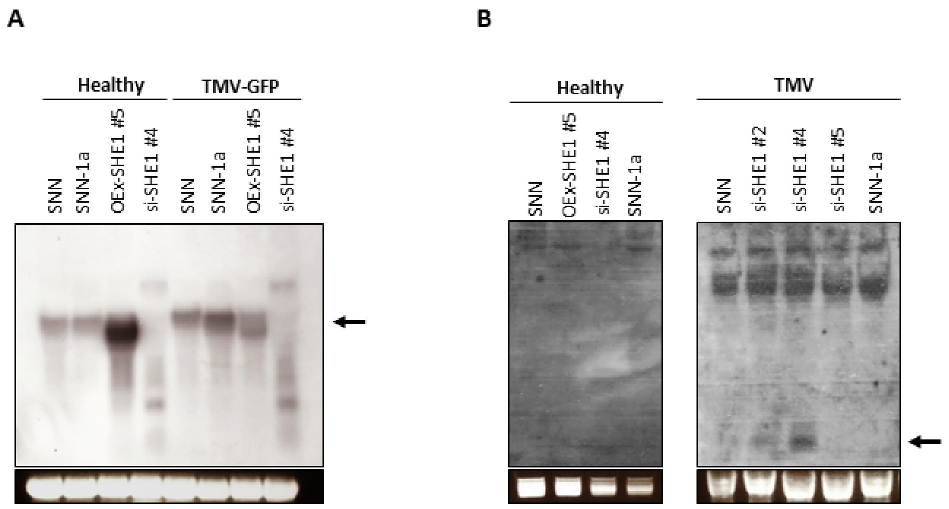

Figure S3. Northern blot hybridization analysis for detection of either (A) SHE1 mRNA or (B) siRNA to SHE1 in nontransformed (SNN) and transgenic tobacco lines, noninoculated or inoculated with either (A) TMV-GFP or (B) TMV. Transgenic tobacco lines include SNN expressing cucumber mosaic virus 1a (SNN-1a), overexpressing SHE1 (Oex-SHE1#5), or silenced for expression of SHE1 (si-SHE1 #2, #4, or #5).

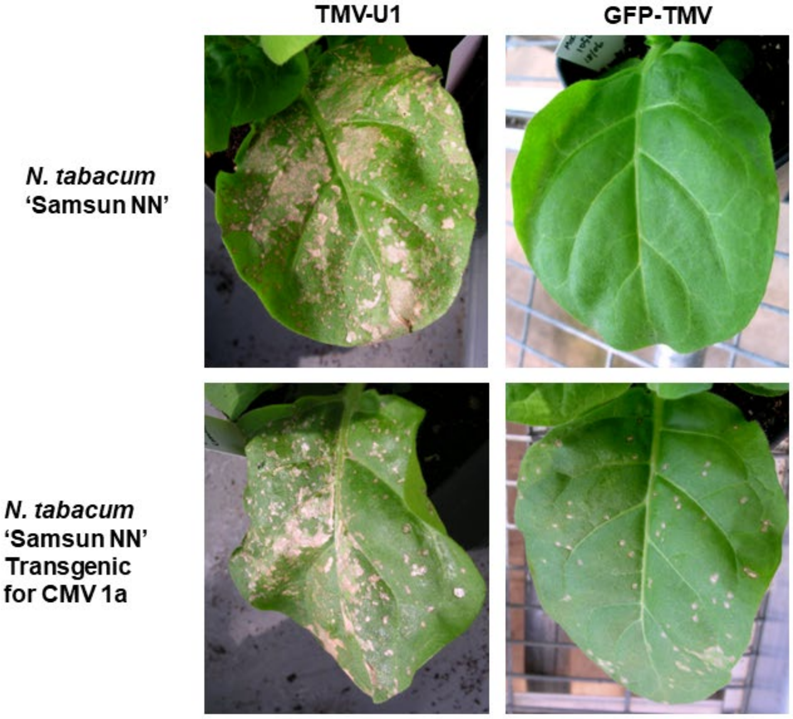

Figure S4. Local response of nontransformed and cucumber mosaic virus (CMV) 1a transgenic *N. tabacum* cv. Samsun NN to infection by either TMV or TMV-GFP.

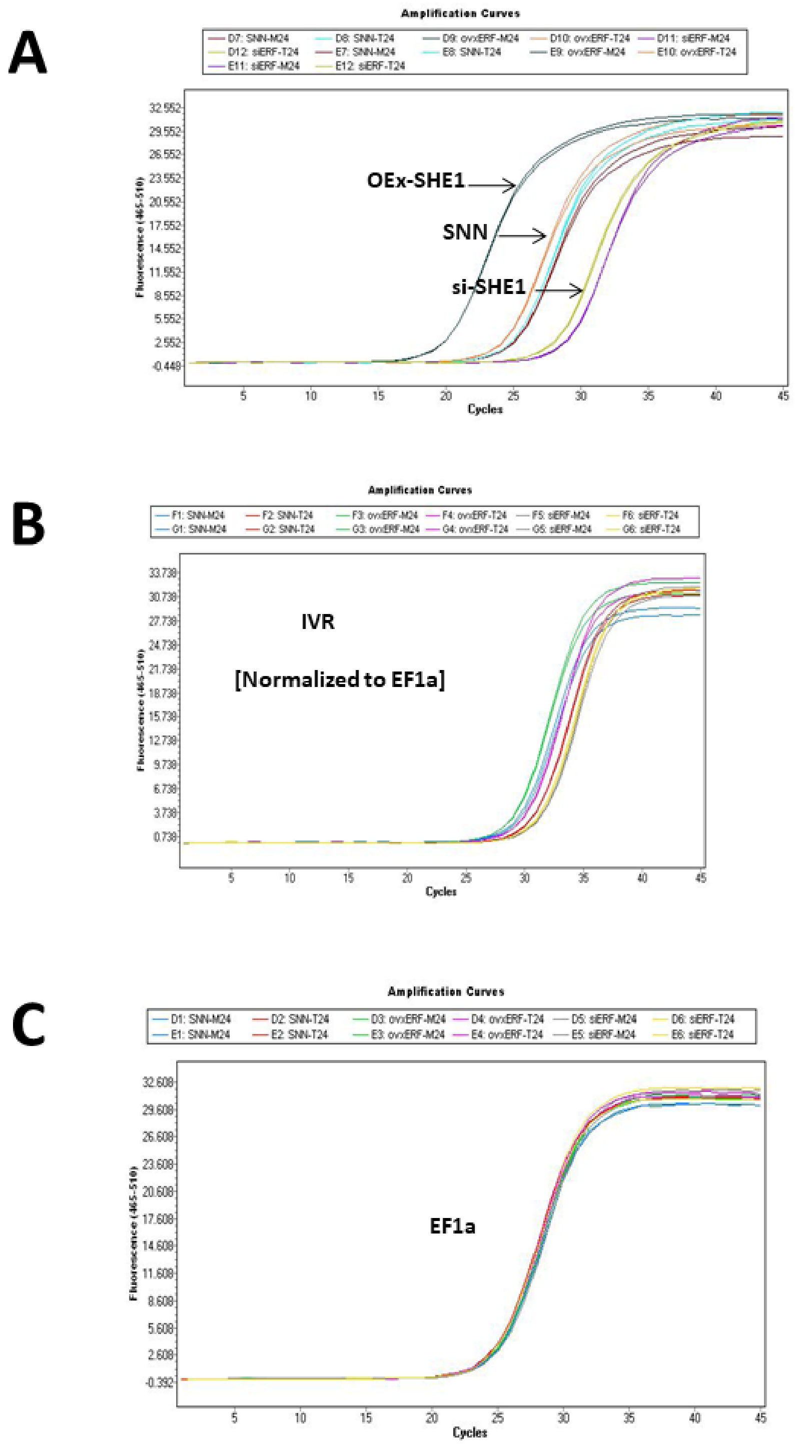

Figure S5. Amplification curves from RT-qPCR analysis of SHE1 and IVR mRNA accumulation in nontransformed (SNN) and transgenic Samsun NN tobacco plants. Plants were transgenic for either overexpression of SHE1 (OEx-SHE1) or silencing of expression of SHE1 (si-SHE1). Plants were inoculated with TMV. Total RNAs from the inoculated leaves were extracted and processed at 24 h post-inoculation, followed by RT-qPCR analysis for the levels of (A) SHE1 mRNAs and (B) IVR mRNAs. The mRNAs from eIF1α were used as a reference standard.

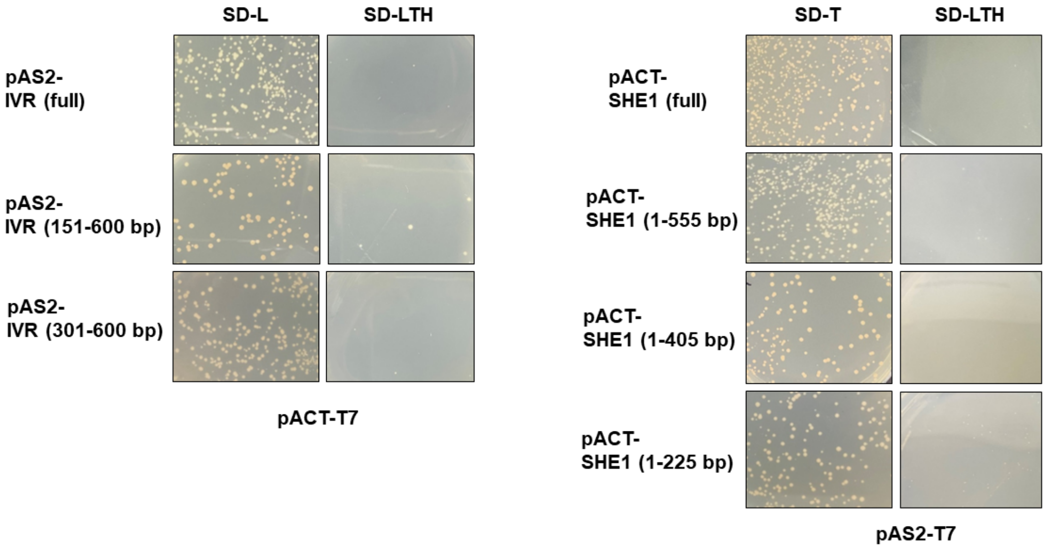

Figure S6. Protein auto-activation test of SHE1 or IVR in yeast cells. Yeast clones expressing either BD-SHE1 or AD-IVR were separately transformed into yeast with empty AD vector (pACT-T7) or empty BD vector (pAS2-T7), respectively. The transformants were plated on SD-L (lacking leucine) or SD-T (lacking tryptophan) medium, respectively. Transformants were not selected when plated on SD-LTH media.

A. IVR: ORF original published nucleotide sequence with errors highlighted (in yellow).

```
GCACGAGTAT TCTTCTCGAT GAATCGACCT GAAGCTGCTG TGGTTGCCTT TAGAGGAGCT   60
CAAGAGTTGA GACCTGATCT TCGTTCTTAT CAAGGTTTAG TACGCTCTTA TTTAGCAATT  120
TCAAAGATCA AGGAGGCACT GCATGCTGCA AGGGAGGCAA TGAAGGCCAT GCCACAGTCA  180
GCAAAAGCTC TAAAATTGGT GGGTGATGTA CATGCTAGTA ATACTAGTGG GAGGGAAAAG  240
GCAAAGAAGT TCTATGAATC TTCACTAAGG CTGGAACCTG GTTATCTTGG AGCAGCGCTA  300
GCCTTGGCAG AACTGCATGT CATTGAAGGT CGAACTGTAG ATGCTGTTAC TCTCCTTGAG  360
CGGTATCTCA AGGATTGGGC TGATGACTCG TTGCATGTTA AGCTTGCCCA AGTATTTGCT  420
GCAACAAATA TGCCACAAGA TGCCTTGTCA CACTACCAGG CAGCACTGAG GATAAATCCC  480
CAAAATGAAG CTGCAAAGAA AGGGTTGGGA GCGCCTGGAG AAGCAGCTCA AGGGAGTAGA  540
TCTGATGCTC CTGAAGAAGA TGAAGAGAAT GATGCTGAGG ATGCTGATGC AGACCAGGAA  600
GAGGCTGAGC TTTTATGATG 620
```

B. IVR: ORF corrected nucleotide sequence with changes highlighted (in turquoise).

```
AGGGAAATAT TCTTCTCGAT GAATCGACCT GAAGCTGCTG TGGTTGCCTT TAGAGGTGCT   60
CAAGAGTTGA GACCTGATCT TCGTTCTTAT CAAGGTTTAG TACGCTCTTA TTTAGCAATT  120
TCAAAGATCA AGGAGGCACT GCATGCTGCA AGGGAGGCAA TGAAGGCCAT GCCACAGTCA  180
GCAAAAGCTC TAAAATTGGT GGGTGATGTA CATGCTAGTA ATACTAGTGG GAGGGAAAAG  240
GCAAAGAAGT TCTATGAATC TTCACTAAGG CTGGAACCTG GTTATCTTGG AGCAGCGCTA  300
GCCTTGGCAG AACTGCATGT CATTGAAGGT CGAACTGTAG ATGCTGTTAC TCTCCTTGAG  360
CGGTATCTCA AGGATTGGGC TGATGACTCG TTGCATGTTA AGCTTGCCCA AGTATTTGCT  420
GCAACAAATA TGCCACAAGA TGCCTTGTCA CACTACCAGG CAGCACTGAG GATAAATCCC  480
CAAAATGAAG CTGCAAAGAA AGGGTTGGΔA GCGCCTGGAG AAGCAGCTCA AGGGAGTAGA  539
TCCTGATGCTC CTGAAGAAGA TGAAGAGAAT GATGTCGAGG ATGCTGATGC AGACCAGGAA 600
GAGGCTGAGC TTTTATGATG 620
```

C. IVR: original published amino acid sequence with errors highlighted (in yellow).

MNRPEAAVVAFRGAQELRPDLRSYQGLVRSYLAISKIKEALHAAREAMKAMPQSAKALKLVGDVHAS
NTSGREKAKKFYESSLRLEPGYLGAALALAELHVIEGRTVDAVTLLERYLKDWADDSLHVKLAQVFAAT
NMPQDALSHYQAALRINPQNEAAKKGLGAPGEAAQGSRSDAPEEDEENDAEDADADQEEAELL_199

D. IVR: revised amino acid sequence derived from the corrected nucleotide sequence highlighted (in turquoise).

MNRPEAAVVAFRGAQELRPDLRSYQGLVRSYLAISKIKEALHAAREAMKAMPQSAKALKLVGDVHAS
NTSGREKAKKFYESSLRLEPGYLGAALALAELHVIEGRTVDAVTLLERYLKDWADDSLHVKLAQVFAAT
NMPQDALSHYQAALRINPQNEAAKKGLERLEKQLKGVDPDAPEEDEENDVEDADADQEEAELL_199

(revised seq)

Figure S7. Original and revised IVR nucleotide (A & B) and putative encoded amino acid (C & D) sequence, respectively. ORF initiation and termination sequences are both in bold and underlined. Changes in either nucleotide sequence described in the text are highlighted and underlined, as well as are changes in amino acid sequence. The 5' nontranslated region of the NC330 cDNA also clone contained sequence differences from the tobacco IVR gene (highlighted).
